# Draft genome sequences of *Pantoea* sp. strains QMID1-QMID4 isolated from the midgut of Japanese honey bee (*Apis cerana japonica*)

**DOI:** 10.1128/MRA.00010-23

**Published:** 2023-07-26

**Authors:** Akihiko Suzuki, Nobuyoshi Nakajima, Yoshiko Sakamoto

**Affiliations:** 1 National Institute for Environmental Studies, Tsukuba, Ibaraki, Japan; University of Maryland School of Medicine, Baltimore, Maryland, USA

**Keywords:** *Pantoea*, honey bee, *Apis cerana japonica*, intestinal bacteria, genomes, carotenoids

## Abstract

We report the draft genome sequences of *Pantoea* sp. strains QMID1-QMID4 that were recovered from the midgut of Japanese honey bee (*Apis cerana japonica*). The strains possess the carotenoid biosynthetic gene cluster. The genome information expands our knowledge of their potential use as probiotics and/or prebiotics in honey bees.

## ANNOUNCEMENT

Members of *Pantoea* have been isolated from various habitats and insects (1, 2) and act as biocontrol agents (3) and as potential honey bee probiotics (4). Here, we report the draft genome sequences of *Pantoea* sp. strains QMID1-QMID4, which were isolated from the midgut of two Japanese honey bees (*Apis cerana japonica*), during the investigation into potentially novel honey bee-beneficial bacteria.

The midguts were carefully removed from the honey bees and then homogenized in sterile phosphate-buffered saline. The homogenate was serially diluted, inoculated onto agar plates containing 50 g L^−1^ sucrose and 10 g L^−1^ dried yeast, and aerobically incubated at 35℃. Genomic DNA (gDNA) was extracted from a single colony by disruption using glass beads and purification using the Qiagen DNeasy Blood and Tissue kit (Qiagen, Hilden, Germany). The libraries for paired-end (2 × 300 bp) sequencing were prepared from the gDNA using NEBNext Ultra II DNA Library Prep Kit for Illumina (New England Biolabs, Ipswich, MA, USA) and then sequenced using MiSeq reagent kit V3 (Illumina, San Diego, CA, USA), generating 1,498,650—1,723,208 raw reads.

FastQC ver. 0.11.9 (5) was used for the quality check of the raw reads. Removal of low quality (quality score <30), adapter, and short reads (<50 bp) using fastp ver. 0.22.0 (6) yielded 1,383,862—1,585,968 high-quality reads. The genome was assembled using SPAdes ver. 3.12.0 (7) with the --careful option. Gene annotation was performed using Prokka ver. 1.14.6 (8). Genome completeness and contamination were determined using CheckM ver. 1.1.3 (9). Default parameters were used except otherwise stated. The detailed genome information is shown in Table 1.

**TABLE 1:** Summarized data on the genome information of *Pantoea* sp. strain QMID1-QMID4

Features	QMID1	QMID2	QMID3	QMID4
No. of raw reads	1,498,650	1,723,208	1,643,400	1,606,114
No. of reads after trimming	1,383,862	1,585,968	1,470,816	1,445,448
Genome size (bp)	4,806,612	4,783,982	4,784,825	4,782,214
G + C content (%)	54.9	55.0	55.0	58.0
No. of contig	114	65	66	53
*N*_50_ contig length (bp)	556,299	461,533	556,299	461,633
No. of protein-coding sequences	4,482	4,461	4,467	4,464
No. of rRNA	17	18	15	15
No. of tRNA	75	75	75	75
No. of tmRNA	1	1	1	1
Genome completeness (%)	99.3	99.3	99.3	99.3
Genome contamination (%)	0.63	0.46	0.26	0.28
RUN accession no.	DRR426820	DRR426821	DRR426822	DRR426823
BioSample	SAMD00568167	SAMD00568168	SAMD00568169	SAMD00568170
Assembled genome accession no.	BSXI01000000	BSXJ01000000	BSXK01000000	BSXL01000000

The average nucleotide identities (ANI) analysis, performed using the ANI calculator software (10), revealed that the genome of strains QMID1-QMID4 showed the highest homology (96.1%) against *P. vagans* LMG 24199^T^ (GenBank accession: GCA_004792415.1). Meanwhile, the values of digital DNA–DNA hybridization (dDDH), calculated using the genome-to-genome distance calculator (11), between the strains QMID1-QMID4 and all *Pantoea*-type strains were less than 70%, which is the proposed boundary for distinguishing between closely related bacteria species (12).

**Fig 1:**
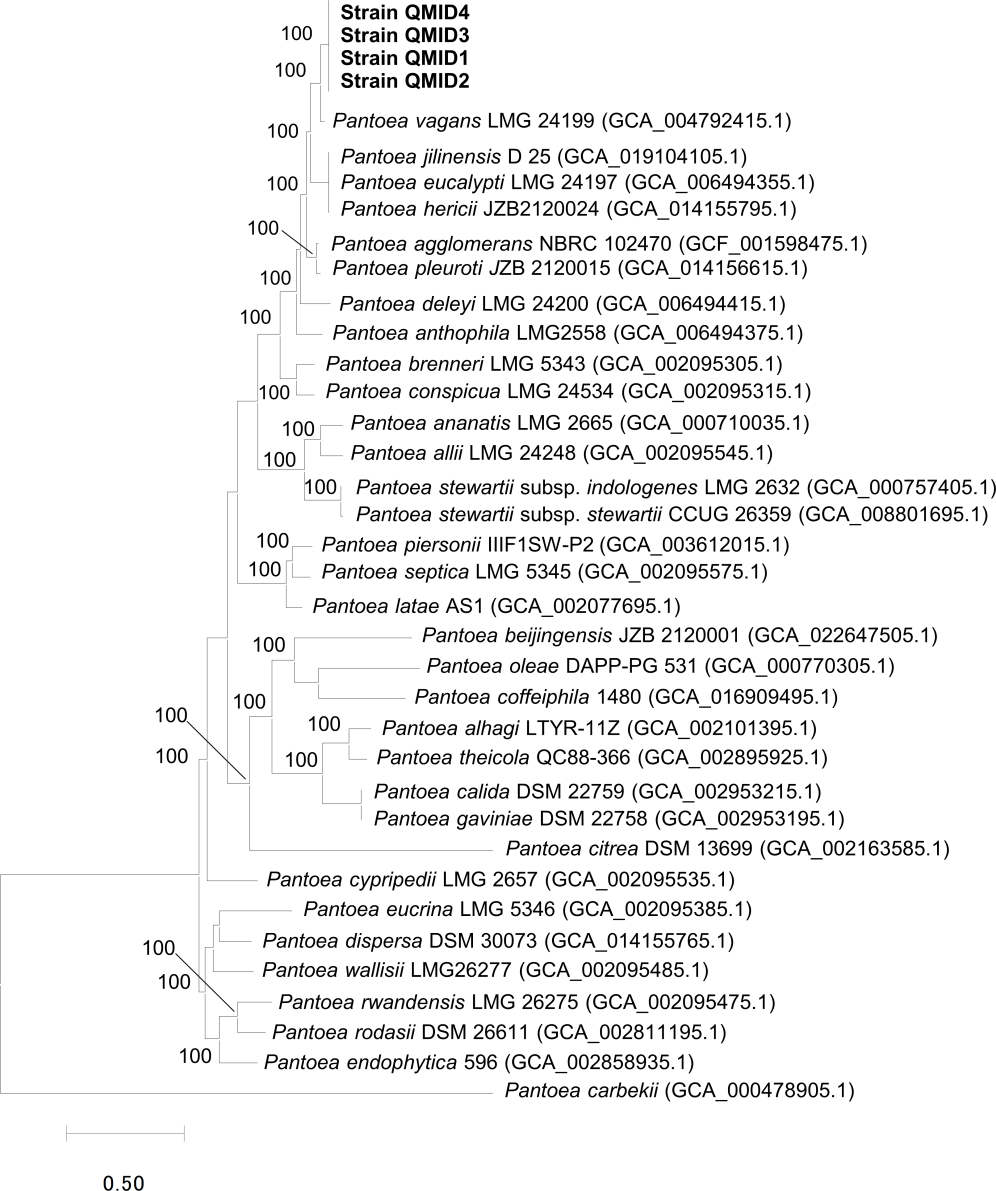
A genome-based maximum likelihood phylogenomic tree constructed based on the nucleotide and amino acid sequences of 360 single-copy genes extracted from the strains QMID1-QMID4 (shown as bold) and available 33 *Pantoea*-type strains using the codon tree in PATRIC pipeline. The numbers in the tree were bootstrap values based on the 100 rounds of rapid bootstrapping option of RAxML. The scale bar represents the evolutionary distance of the nucleotide substitutions per site.

A concatenated nucleotide and protein sequences of 360 single-copy genes extracted from the strains in this study and the available genomes of *Pantoea*-type strains were aligned using the Codon_align function of BioPython (13) and MUSCLE (14), respectively. A maximum likelihood phylogenomic tree was constructed using RAxML (15) with the 100 rounds of rapid bootstrapping option for calculating the branch support values (16) in the codon tree of the PATRIC pipeline (17). The tree showed that the strains QMID1-QMID4 formed a separate branch from the *Pantoea*-type strains (Fig. 1). Overall, the results of dDDH and the phylogenetic tree confirmed that the strains QMID1-QMID4 are potentially novel species in the genus *Pantoea*.

The antiSMASH software ver. 6.0 (18) detected the carotenoid biosynthetic genes (*crtE*, -*X*, -*Y*, -*I*, -*B*, and -*Z*) (19) in the strains QMID1-QMID4. The homologies of deduced amino acid sequences against each of the sequences in UniProt database (20) were over 95.1%. This report will expand our knowledge of probiotics and prebiotics in honey bees.

## Data Availability

The draft genome sequences for the strains QMID1-QMID4 have been deposited at DDBJ Sequence Read Archive (SRA) database under the accession number: DRA015411 (RUN number: DRR426820-DRR426823, BioProject: PRJDB15005), and assembled genome data are available under the accession numbers: BSXI01000000, BSXJ01000000, BSXK01000000, and BSXL01000000, respectively, as shown in Table 1.
